# A new strategy improving TB diagnosis: stratified urine LAM test based on lymphocyte counts

**DOI:** 10.3389/fcimb.2025.1498651

**Published:** 2025-02-20

**Authors:** Hongzhi Li, Xian Gao, Dandan Liu, Zhe Li, Jing Li

**Affiliations:** ^1^ Department of TB Diseases, Affiliated Infectious Diseases Hospital of Zhengzhou University, Zhengzhou, China; ^2^ Department of TB Diseases, Henan Infectious Diseases Hospital, Zhengzhou, China; ^3^ Department of TB Diseases, The Sixth People’s Hospital of Zhengzhou, Zhengzhou, China

**Keywords:** tuberculosis, lipoarabinomannan, lymphocyte, diagnosis, biomarker

## Abstract

**Background:**

Traditional lipoarabinomannan tests have limited sensitivity in HIV-negative individuals. Our aims were to compare chemiluminescence-based LAM (AIMLAM) and other diagnostic modalities in HIV-negative patients and to explore whether lymphocyte counts impact the sensitivity and costs of AIMLAM.

**Methods:**

This is a prospective, cross-sectional, diagnostic accuracy study. Participants underwent testing with sputum acid-fast bacilli, sputum culture, GeneXpert, and AIMLAM. Their diagnostic efficiency and cost-effectiveness alone or under different lymphocyte count categories was evaluated.

**Results:**

Using MRS as a reference, the sensitivities of different diagnostic methods were as follows: sputum smear 27.43%, sputum culture 45.13%, GeneXpert 74.34%, and AIMLAM 71.68%. Patients with lymphocyte counts <0.8 × 10^9^/L were significantly more likely to have a positive AIMLAM result (OR = 9.431, 95% CI: 2.659–33.447, *P* = 0.001). The sensitivity of AIMLAM in patients with lymphocyte counts <0.8 × 10^9^/L reached 93.02%. The overall cost of AIMLAM to detect a positive TB case was $129.82, lower than sputum culture ($136.10) and GeneXpert ($180.27). For patients with lymphocyte counts <0.8 × 10^9^/L, the cost of AIMLAM was further reduced to $67.84 (a 47.74% decrease), which was lower than GeneXpert ($111.65) and sputum culture ($94.28).

**Conclusion:**

AIMLAM showed promising diagnostic performance in HIV-negative patients. Stratifying patients using lymphocyte cell counting lifted the sensitivity and lowered the cost of LAM, offering a novel diagnostic strategy for tuberculosis in resource-limited settings.

## 1 Introduction

Tuberculosis (TB) is an infectious disease caused by *Mycobacterium tuberculosis* (Mtb). It is ranked the second most deadly infectious disease after COVID-19. There were a total of 10.6 million cases of LTBI and 1.6 million deaths due to TB worldwide (World Health Organization, 2023). Among LTBI, 5%–10% would progress to active tuberculosis (ATB) (Ding et al., 2022).

For decades, sputum-based tests have remained the primary methods for diagnosis. However, only 75% of symptomatic patients and 10% of asymptomatic patients are able to produce sputum by themselves (Lissouba et al., 2021). Therefore, the WHO called for new non-sputum tests such as the urine test. The urine lipoarabinomannan (LAM) test is the most popular non-sputum test for TB diagnosis. LAM is a component of the cell envelope of Mtb (De et al., 2024), mediating host immune responses (Fukuda et al., 2013). After being released into the blood by Mtb, LAM reaches the kidneys and subsequently filters through the glomerular basement membrane into the urine (Bulterys et al., 2019). This lays the theoretical foundation for the detection of LAM in urine. The LAM test is promising for tuberculosis diagnosis, especially in people with HIV and in disseminated tuberculosis patients (Gupta-Wright et al., 2016).

Currently, the only commercially available LAM test kit is Alere’s Determine TB LAM (AlereLAM), based on colloidal gold, with a limit of detection (LOD) of 500 pg/mL (García et al., 2019). The sensitivity of AlereLAM is 10.8%–18% in the HIV-negative population (Minion et al., 2011; Broger et al., 2020a) and 40% in people with HIV (Huerga et al., 2023). While another product Fujifilm SILVAMP TB LAM (FujiLAM) has been extensively researched, its variability between different lot numbers has hindered its application (Huerga et al., 2023). The sensitivity of FujiLAM in people with HIV has reached 70.7% (Broger et al., 2020a) and has been reported at 53% in the HIV-negative population (Broger et al., 2020a), thanks to the lowered LOD (30 pg/mL) (Broger et al., 2019). Reducing the LOD may be the main approach to improving sensitivity while ensuring stability and specificity. The new LAM test, based on chemiluminescence and urine concentration (AIMLAM), significantly improves the sensitivity of the LAM test. Studies have shown a sensitivity of 55% in HIV-negative populations. This will greatly enhance the diagnosis of TB in the general population.

In addition to lowering LOD to enhance sensitivity, employing immune status stratification strategies can also improve sensitivity. Mtb can replicate extensively within the body without sufficient immune capacity (Chandra et al., 2022), leading to an increased circulation of LAM. Immune suppression also reduces the formation of antigen–antibody complexes, resulting in more free LAM present in the circulation (Lawn, 2012). Therefore, in patients with immune deficiency and suppressed immunity, the concentration of LAM in the urine is much higher (Paris et al., 2017). The WHO recommends AlereLAM only to be used in people with HIV with CD4^+^ T cells less than 200/mm^3^. This stratification strategy based on CD4^+^ T cells is based on immunity’s critical role in the mechanism of LAM’s emission to urine. However, in the general HIV-negative population, routine CD4^+^ T-cell testing is not performed and there is a lack of immune stratification markers to fully assess the sensitivity and specificity of the LAM test.

In contrast, a complete blood count (CBC) is routinely performed, is cost-effective, and can provide an initial assessment of a patient’s immune function (Gulati et al., 2022). CD4^+^ T cells account for 23.78%–51.07% of lymphocytes (Silva et al., 2001). Studies have shown that both CD4^+^ T-cell and CD8^+^ T-cell lymphocytes are involved in the immune response against tuberculosis (Silva et al., 2001), and previous research has reported lower lymphocytes in TB patients (Luo et al., 2021). Lymphocyte counts may have similar effects to CD4^+^ T-cell counts.

This study aims to explore whether lymphocyte counts can be used for immune stratification of HIV-negative TB patients. Additionally, the study will analyze the sensitivity and specificity of AIMLAM in tuberculosis patients with different immune stratifications and compare it with other methods.

## 2 Methods

### 2.1 Study design and participants

In this prospective, cross-sectional, double-blind diagnostic accuracy study, presumed tuberculosis patients from the Affiliated Infectious Diseases Hospital of Zhengzhou University from November 2023 to February 2024 were included. Collection of samples and diagnosis were performed before treatment. The sample size was calculated using PASS 21.0 based on the sensitivity and specificity of the AIMLAM test. According to our previous research, the prevalence of microbiological reference standard (MRS) TB in presumed TB patients was approximately 40%, the lowest acceptable sensitivity was 50%, and the expected sensitivity was 70%. Both acceptable and expected sensitivity were 95% and *α* was 0.05. Power (1 − *β*) was set at 0.9, and the dropout rate was 10% (Gao et al., 2024; Huang et al., 2023). Results showed that we needed to include 180 presumed cases of TB.

This study followed the Standards for Reporting of Diagnostic Accuracy (STARD) guideline. The study adhered to the Helsinki Declaration and was approved by the Medical Ethics Committee of the Sixth People’s Hospital of Zhengzhou. The ethics number is IEC-KY-2023-48.

### 2.2 Participants

We included presumptive TB patients meeting the following criteria: 1) aged ≥18, men and women; 2) with symptoms of TB (cough of at least 2 weeks, unexplained fever, weight loss, night sweats); and 3) able to produce sputum and collected urine. Patients were excluded if they 1) had HIV and 2) were receiving tuberculosis treatment.

### 2.3 Definition

The definition of definite TB and possible TB was based on the “Diagnosis for pulmonary tuberculosis (WS 288-2017)” (Commission, 2018) and the WHO tuberculosis guideline (World Health Organization, 2022). Definite TB was defined as Active tuberculosis (ATB), through TB culture or acid fast bacillusor or GeneXpert. Possible TB was defined as patients not meeting the criteria of definite TB but had X-ray positive, and they 1) had symptoms and signs or 2) were TST or IGRA or TB antibody positive. Non-TB was defined as patients not eligible for definite or possible TB.

Microbiological reference standard (MRS) is a benchmark diagnostic criterion based on direct microbiological evidence of infection. In tuberculosis diagnostics, MRS typically includes methods such as sputum smear microscopy, culture, and nucleic acid amplification tests (e.g., GeneXpert), which detect the presence of *Mycobacterium tuberculosis* directly (Shreffler and Huecker, 2020).

Composite reference standard (CRS) involves a composite of clinical assessments, including patient history, physical examinations, radiological findings, and sometimes histopathological evidence, to diagnose an infection (Shreffler and Huecker, 2020).

### 2.4 Sample collection

Sputum, urine, and blood were collected before any treatment was involved. Sterile, dry containers were used to collect three samples of sputum, including those obtained immediately, at night, and in the early morning. These were combined into one specimen, dissolved thoroughly in 5 mL of saline solution, and stored at 4°C for examination. Approximately 5 mL of midstream urine from the patient was collected in the morning and stored at 4°C for examination. Approximately 5 mL of fasting venous blood was collected and stored at room temperature.

### 2.5 Testing procedures

Urinary LAM detection was conducted using AIMLAM kits (Leide Biosciences Co., Ltd, Guangzhou, China) according to the user’s manual (Zhang et al., 2023; Gao et al., 2024; Huang et al., 2023). The performers were blinded to the clinical information of the participants. This kit utilizes a chemiluminescent immunoassay to detect LAM in urine. LAM-specific antibodies immobilized on magnetic beads capture LAM, forming a complex of magnetic bead–antibody–antigen. Subsequently, the complex binds to a luminescent label, resulting in the formation of a magnetic bead–antibody–antigen–aminoluciferin label complex. Following separation and washing, a pre-triggering solution and a triggering solution were introduced to the reaction mixture, with the level of LAM in the test sample being directly proportional to the relative light unit value obtained.

The acid-fast stain (AFB) and microscopy were performed on sputum based on the Ziehl-Neelsen stain (van Deun et al., 2008). Sputum culture was performed according to protocols (Hanna et al., 1999) previously established based on the BACTEC™ MGIT™ 960 TB system (BD Diagnostic Systems, Sparks, MD, USA). GeneXpert was performed on 1.0 mL of sputum sample according to the user’s instruction (Cepheid, Inc., Sunnyvale, CA, USA) (Giang Do et al., 2015). The performers of the above tests were masked to the AIMLAM results and clinical information of the participants.

### 2.6 Assessment of blood parameters

White blood cell count (WBC), granulocyte count, lymphocyte count, erythrocyte sedimentation rate (ESR), procalcitonin (PCT), and C-reactive protein (CRP) were determined using a fully automated hematology analyzer (Mindray BC-5180 CRP).

### 2.7 Statistics

All statistical analyses were conducted using SPSS 26.0. The diagnostic accuracy of AIMLAM compared with MRS and CRS was calculated. Continuous data were transformed into categorical data according to the following methods. Age was categorized using two cutoff values: 30 and 60 years. Blood cell count, ESR, PCT, and CRP were categorized based on commonly used clinical reference standards, as follows: WBC, 4–10 × 10^9^/L; lymphocytes, 0.8–4 × 10^9^/L; neutrophils, 1.8–6.3 × 10^9^/L; monocytes, 0.1–0.6 × 10^9^/L; ESR, 0–10 mm/h for men and 0–20 mm/h for women; CRP, 0–10 mg/L; and PCT, 0.5 ng/L. Because there were only three cases (1.21%) and one case (0.88%) in the high lymphocyte count group with LAM positive (LAM+) or LAM negative (LAM−), we grouped patients with normal and high lymphocyte counts into the ≥0.8 × 10^9^/L category. Categorical data were expressed as frequencies and percentages.

### 2.8 Calculating sensitivity and specificity

Chi-square tests or Fisher’s exact probability method was used for analysis. The sensitivity and specificity of each detection method were calculated based on MRS and CRS (Shreffler and Huecker, 2020; Kraef et al., 2022; Broger et al., 2020b).

Sensitivity = true positive cases/(true positive cases + false negative cases) × 100%.
Specificity = true negative cases/(true negative cases + false positive cases) × 100%.

### 2.9 Logistic regression analysis

MRS-positive patients were divided into two groups: LAM+ and LAM−. General data and blood indicators of the two groups were analyzed for differences. Logistic regression was employed to examine the association between lymphocyte count and AIMLAM. Model 1 included only lymphocyte count as the independent variable. Model 2 was adjusted for gender and age. Model 3 was further adjusted for factors in model 2 as well as diabetes and autoimmune diseases. Model 4 was additionally adjusted for variables in model 3 and blood indicators showing statistically significant differences between LAM− and LAM+ individuals. Fagan nomography was used to demonstrate the clinical utility of AIMLAM in diagnosing TB patients from presumed TB patients.

### 2.10 Calculation of average cost per detected TB patient

The cost per detected TB patient was calculated as the total testing cost divided by the number of true positive cases. For all presumed TB patients, the average cost per person was determined using the formula (Aliyu et al., 2014; Gao et al., 2024):

(1) Average cost for all presumed TB patients:

Average cost = (total number of individuals × unit price)/number of true positive cases

For patients with low lymphocyte counts, the average cost per person was also calculated.

The prices of all test reagents in this article are only applicable to the time of testing in this study. Currently, there is no confirmed price for AIMLAM. All costs were calculated based on the average exchange rate for the year 2023 (1 USD = 7.075 CNY), with the exchange rate sourced from the IRS website (https://www.irs.gov/individuals/international-taxpayers/yearly-average-currency-exchange-rates). A significance level of *P* <0.05 was considered statistically significant.

## 3 Results

### 3.1 Demographic characteristics

**Figure 1:**
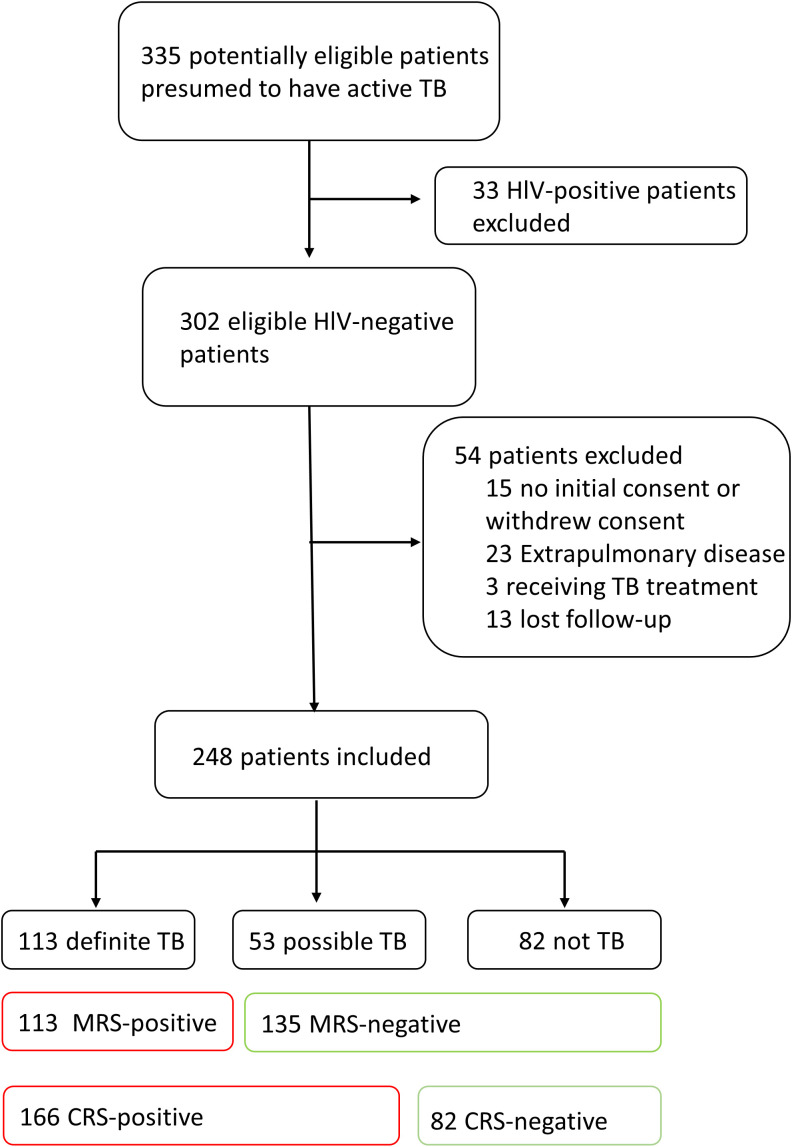
Flow diagram of patient selection.

Initially, 335 patients were enrolled. Thirty-three people with HIV were excluded, 15 were excluded for not providing consent or withdrawing consent, 23 were excluded for extrapulmonary disease, 3 were excluded for receiving TB treatment, and 13 were excluded for lost follow-up. Finally, a total of 248 patients were included in the analysis. Among them, 113 were definite TB cases, 53 were possible TB cases, and 135 were non-TB (**Figure 1**). There were 131 men and 117 women. Fifty-three were less than 30 years old, 118 were 30–60 years old, and 77 were older than 60 years. Fifty-three cases (21.47%) had diabetes mellitus (DM), and 24 cases (9.68) had a history of TB. Sixty-one cases (24.6%), 184 cases (74.19%), and 3 cases (1.21%) had low, normal, and high lymphocyte counts, respectively. The distribution of sex, DM, monocytes, lymphocytes, ESR, PCT, and CRP was significantly different between groups (**Supplementary Table 1**).

### 3.2 Diagnostic performance of LAM

**Figure 2:**
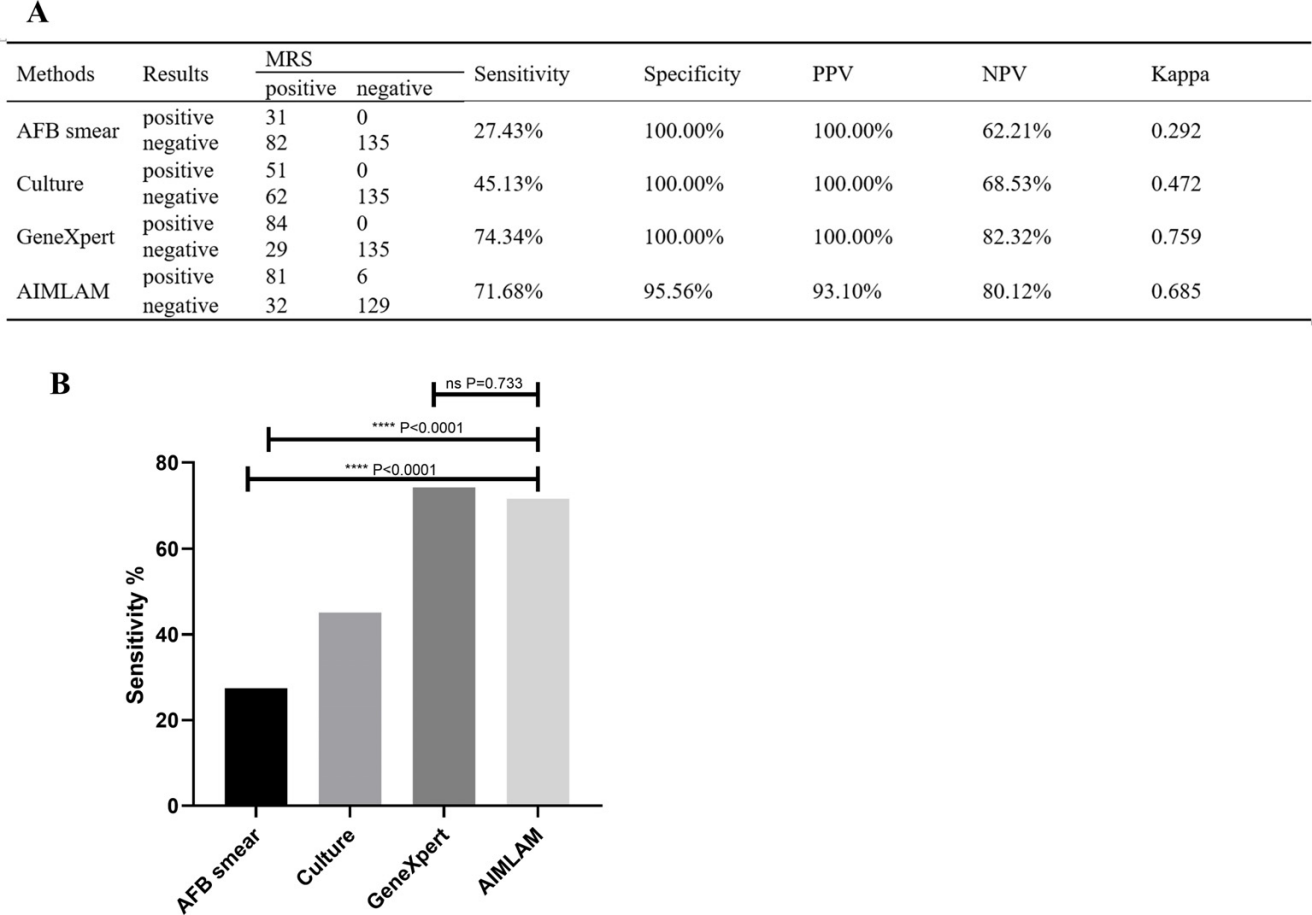
**(A)** Diagnostic performance of the four methods compared to MRS. **(B)** Sensitivity difference analysis of the four methods based on MRS. AFB, acid-fast bacillus; LAM, lipoarabinomannan; MRS, microbiological reference standard; PPV, positive predictive value; NPV, negative predictive value. ****, *P* < 0.0001; ns, *P* > 0.05. Sensitivity = true positive cases/(true positive cases + false negative cases) × 100%; Specificity, true negative cases/(true negative cases +false positive cases) × 100%; PPV, true positive cases/(true positive cases + false positive cases) × 100%; NPV, true negative cases/(true negative cases + false negative cases) × 100%.

Using MRS, the sensitivity rates of AFB, culture, GeneXpert, and AIMLAM were 27.43%, 45.13%, 74.34%, and 71.68%, respectively. The sensitivity of AIMLAM was significantly higher than that of the AFB smear and culture but showed no significant difference compared to GeneXpert. The specificity of AFB, culture, and GeneXpert was 100%, as they compromised the MRS (**Figure 2B**). The specificity of AIMLAM was 95.56% (**Figure 2A**). Using CRS, the sensitivity rates of the AFB smear, culture, GeneXpert, and AIMLAM were 18.67%, 30.72%, 50.60%, and 51.20%, respectively. The specificity of the AFB smear, culture, and GeneXpert was 100%. The specificity of AIMLAM was 97.56% (**Supplementary Figure 1**).

### 3.3 Analysis of the risk factors of AIMLAM

Among the 113 definite TB patients, 81 were LAM-positive. Age, sex, comorbidity, and other serum variables were not statistically different between LAM+ patients and LAM− patients. The proportion of low neutrophils, low lymphocytes, high ESR, high PCT, and high CRP in the LAM+ TB group was significantly higher than that in the LAM− TB group. (**Supplementary Table 2**). In the unadjusted model (model 1), patients with low lymphocytes were more likely to have positive LAM results (OR = 9.431, 95% CI: 2.659–33.447, *P* = 0.001). When adjusted for sex and age (model 2), the OR was 9.562 (95% CI: 2.476–36.928, *P* = 0.001). When adjusted for factors in model 2 plus DM and TB history (model 3), the OR was 10.021 (95% CI: 2.555–39.297, *P* = 0.001). When adjusted for factors in model 3 plus neutrophils, ESR, and CRP (model 4), the OR was 5.992 (95% CI = 1.421–25.262, *P* = 0.015). Among the 135 MRS− cases, 6 were LAM positive. None of the factors listed were significantly different between LAM+ and LAM− patients (**Table 1**).

**Table 1:** Results of logistics regression.

Variables	MRS+				MRS−			
	*β*	OR	95% CI	*P*	*β*	OR	95% CI	*P*
Model 1								
Lymphocytes (low)	2.244	9.431	2.659–33.447	0.001	0.276	1.318	0.145–11.973	0.806
Model 2								
Lymphocytes (low)	2.258	9.562	2.476–36.928	0.001				
Sex (male)	0.023	1.023	0.409–2.559	0.961				
Age (30–60 years)	Ref	Ref	Ref	Ref	Ref	Ref	Ref	Ref
Age (<30 years)	−0.373	0.688	0.245–1.933	0.478				
Age (>60 years)	−0.269	0.764	0.234–2.492	0.655				
Model 3								
Lymphocytes (low)	2.305	10.021	2.555–39.297	0.001				
Age (30–60 years)	Ref	Ref	Ref	Ref	Ref	Ref	Ref	Ref
Age (<30 years)	−0.272	0.762	0.257–2.261	0.624				
Age (>60 years)	−0.179	0.836	0.252–2.778	0.770				
Sex (male)	0.025	1.025	0.4–2.626	0.959				
DM (yes)	–0.212	0.809	0.248–2.644	0.726				
TB history (yes)	–0.891	0.410	0.099–1.703	0.220				
Model 4								
Lymphocytes (low)	1.790	5.992	1.421–25.262	0.015				
Age (30–60 years)	Ref	Ref	Ref	Ref	Ref	Ref	Ref	Ref
Age (<30 years)	−0.318	0.727	0.224–2.363	0.596				
Age (>60 years)	–0.275	0.759	0.206–2.793	0.679				
Sex (male)	0.040	1.040	0.383–2.827	0.938				
DM (yes)	−0.145	0.865	0.25–2.999	0.819				
TB history (yes)	−1.045	0.352	0.079–1.574	0.172				
Neutrophils (low)	1.167	3.213	0.623–16.577	0.163				
ESR (high)	−0.001	0.999	0.23–4.336	0.999				
PCT (high)	0.439	1.551	0.375–6.412	0.545				
CRP (high)	0.678	1.971	0.459–8.456	0.361				

MRS, microbiological reference standard; ESR, erythrocyte sedimentation rate; PCT, procalcitonin; CRP, C-reactive protein.

### 3.4 Comparison of the diagnostic performance of AIMLAM between different lymphocyte count categories

All presumed TB patients were grouped into <0.8 × 10^9^/L (64 out of 248) and ≥0.8 × 10^9^/L (184 out of 248) according to their lymphocyte counts. In the <0.8 × 10^9^/L group, the sensitivity rates were 32.56%, 44.19%, 81.40%, and 93.02% for AFB, culture, GeneXpert, and AIMLAM, respectively. In the ≥0.8 × 10^9^/L group, the sensitivity rates were 24.29%, 45.71%, 70.00%, and 58.57%, respectively. The sensitivity of AIMLAM in low lymphocyte patients was much higher than that in high lymphocyte patients (*P* < 0.001) (**Table 2**).

**Table 2:** Diagnostic performance in different lymphocyte groups.

		<0.8 × 10^9^/L			≥0.8 × 10^9^/L			*P*
Methods	Results	MRS		Sensitivity	MRS		Sensitivity	
		Positive	Negative		Positive	Negative		
AFB smear	Positive	14	0	32.56%	17	0	24.29%	0.339
	Negative	29	21		53	114
Culture	Positive	19	0	44.19%	32	0	45.71%	0.874
	Negative	24	21		38	114
GeneXpert	Positive	35	0	81.40%	49	0	70.00%	0.178
	Negative	8	21		21	114
AIMLAM	Positive	40	1	93.02%	41	5	58.57%	<0.001
	Negative	3	20		29	109

Sensitivity = true positive cases/(true positive cases + false negative cases) × 100%.

AFB, acid-fast bacillus; LAM, lipoarabinomannan; MRS, microbiological reference standard; PPV, positive predictive value; NPV, negative predictive value.

The Fagan nomogram was employed to evaluate the clinical value of AIMLAM in diagnosing TB. The Fagan nomogram showed posttest positive and negative probabilities of 93% and 20%, respectively, under a pretest probability set at 46% (**Supplementary Figure 2**).

### 3.5 Evaluating the cost-effectiveness of TB diagnosis across different lymphocyte levels (<0.8 × 10^9^/L and ≥0.8 × 10^9^/L)

For the cost-effectiveness analysis, without considering lymphocyte counts, the cost to detect a positive TB case was $48.40, $136.10, $180.27, and $129.82 for the AFB smear, culture, GeneXpert, and AIMLAM, respectively. After stratifying by lymphocyte counts, for patients with lymphocyte counts <0.8 × 10^9^/L, AIMLAM testing emerged as the most cost-effective option (excluding the AFB smear), achieving a sensitivity of 93% and a specificity of 95%. In this group, the cost of AIMLAM to detect a positive TB case decreased to $67.84 (a 47.74% reduction), which was lower than GeneXpert ($111.65, a 38.06% reduction) and culture ($94.28, a 30.73% reduction). Conversely, for patients with lymphocyte counts ≥0.8 × 10^9^/L, the difficulty of achieving a positive detection increased, and the most cost-effective diagnostic method was the AFB smear, followed by culture, AIMLAM, and finally GeneXpert (**Table 3**).

**Table 3:** Price of detecting a TB case (US $).

Methods	Unit price ($)	True positive (*N*)	Total (*N*)	Overall ($)	Lymphocytes ($)					
					<0.8 × 10^9^/L (change)			≥0.8 × 10^9^/L (change)		
					True positive (*N*)	Total (*N*)	Overall ($)	True positive (*N*)	Total (*N*)	Overall ($)
AFB smear	6.05	31	248	48.40	14	64	27.66 (−42.85%)	17	184	65.48 (35.29%)
Culture	27.99	51	248	136.10	19	64	94.28 (−30.73%)	32	184	103.44 (24.00%)
GeneXpert	61.06	84	248	180.27	35	64	111.65 (−38.06%)	49	184	229.29 (27.20%)
AIMLAM	42.40	81	248	129.82	40	64	67.84 (−47.74%)	41	184	190.28 (46.57%)

Average cost = (total number of individuals × unit price)/number of true positive cases.

Change rate = (Overall (post-lymphocyte stratification) – Overall (pre-lymphocyte stratification))/Overall (pre-lymphocyte stratification) * 100%.

AFB, acid-fast bacillus; LAM, lipoarabinomannan.

This stratification by lymphocyte counts highlights the optimal sequence of diagnostic methods for minimizing costs while maintaining diagnostic accuracy, offering valuable guidance for clinical decision-making. Since CBC testing is routinely performed in clinical practice, it does not contribute additional costs, further enhancing the practical utility of this approach.

## 4 Discussion

The main finding of this study was that the sensitivity of the LAM test in the HIV-negative population was 71.68% (MRS), which is comparable to GeneXpert and higher than AFB and culture. It is also higher than that reported in previous studies, mainly due to the reference standard used in these studies which was CRS (Gao et al., 2024; Zhang et al., 2023; Huang et al., 2023). The sensitivity of LAM was 51.2% when compared to the CRS, consistent with previous reports (Gao et al., 2024; Zhang et al., 2023; Huang et al., 2023). LAM is a tuberculosis pathogenetic test, and the accuracy of AIMLAM in diagnosing ATB is more accurately reflected by using MRS as a reference standard. Lowering the LOD can help improve detection rates (Huerga et al., 2023; Kraef et al., 2022; Broger et al., 2020b). This study also demonstrated that when the LAM detection limit was lowered to 1 pg/mL (Gao et al., 2024), the sensitivity increased from the original 10.8% (Huerga et al., 2023; Broger et al., 2020a) to 51.2% (reference CRS).

It has been extensively shown that urinary LAM concentration is influenced by immune function and immunosuppression, with bacterial proliferation and a subsequent increase in urinary LAM concentrations (Gupta-Wright et al., 2016). This suggests that, in addition to lowering the LOD to improve sensitivity, researchers can also increase LAM sensitivity by identifying and targeting immunosuppressed patients. The WHO recommends immunostratifying patients by CD4^+^ T-cell count to analyze the diagnostic efficacy of LAM. The general sensitivity of AlereLAM was 9%–27% (Peter et al., 2016; Nakiyingi et al., 2014), and it increased dramatically in patients with CD4^+^ T cells less than 200/mm^3^ and 100/mm^3^ (Peter et al., 2016), which means that lower immunity leads to higher sensitivity.

The lymphocyte count encompasses CD4^+^ T cells, CD8^+^ T cells, and B lymphocytes, all of which play critical roles in the immune response against TB infection (An et al., 2022). For HIV-negative patients, CD4^+^ T-cell counts are not routinely measured, and lymphocyte count, as part of CBC, serves as the most convenient method to assess the immune status of patients. Studies have indicated a decline in lymphocyte count among TB patients in the HIV-negative population (Li et al., 2023; Wang et al., 2015). In this study, we discovered that populations with a low lymphocyte count are more likely to be tested positive in the MRS+ population than those with normal and high lymphocyte counts (OR = 9.431, 95% CI: 2.659–33.447), consistent with previous reports (Li et al., 2023). It is suggested that lymphocyte count might serve as a viable surrogate marker for CD4^+^ T cells specifically in HIV-negative individuals. In this study, the sensitivity of conventional TB diagnostic tests such as AFB smear, culture, and GeneXpert was notably higher in individuals with low lymphocyte counts compared to those with high lymphocyte counts. Remarkably, the sensitivity of the AIMLAM test was particularly noteworthy at 93.02%, surpassing that of other traditional tests. This heightened sensitivity associated with the LAM test in individuals with low lymphocyte counts is of paramount clinical significance. It implies that incorporating LAM testing into routine diagnostic protocols can substantially enhance the detection rate of TB patients with low lymphocyte counts, thereby mitigating missed diagnoses and treatment delays, ultimately improving clinical outcomes.

Moreover, the use of CBC, which includes lymphocyte counting, for immunostratification in TB diagnosis offers significant potential for cost reduction. Our study demonstrated that in patients with low lymphocyte counts (<0.8 × 10^9^/L), the cost of detecting one TB case decreased by 30.73% to 47.74% across various diagnostic modalities. Specifically, the cost for AIMLAM testing was reduced to $67.84, making it the second most cost-effective option after smear and significantly more economical than culture and GeneXpert. This dual benefit highlights the practical value of lymphocyte count-based immunostratification as a cost-effective diagnostic tool, particularly in resource-limited settings where the majority of TB cases occur (Organization, 2023). Importantly, CBC is a routine test with minimal additional cost, making it a practical option for widespread implementation.

In contrast, CD4 T-cell testing, while offering more specific immunological stratification, is rarely performed in HIV-negative populations due to its high cost (approximately $40 per test) and lack of routine applicability in this demographic. These factors limit its feasibility as a large-scale screening tool in non-HIV TB diagnosis. For patients with low lymphocyte counts identified via CBC, AIMLAM testing should be prioritized as a supplementary method alongside gold-standard diagnostics such as smear and culture, offering an optimal balance of cost-effectiveness and diagnostic accuracy.

For patients with lymphocyte counts ≥0.8 × 10^9^/L, AIMLAM continues to demonstrate higher sensitivity compared to smear and culture, maintaining its diagnostic value. Although its cost-effectiveness is reduced in this group, it remains a valuable diagnostic modality with potential for broader applicability. Notably, if the cost of LAM testing were to decrease to $30 in the future, the cost of detecting a positive TB case in patients with low lymphocyte counts could be reduced further to $48. This would make AIMLAM not only the most cost-effective option but also more economical than the AFB smear, solidifying its role as a preferred diagnostic method in resource-limited settings.

Our study has limitations. On the one hand, the sample size for patients with low lymphocyte counts was relatively small. Larger sample sizes and multicenter trials are needed to validate the conclusion of high sensitivity in this subgroup. On the other hand, we did not compare the specific LAM test used in our study with the one recommended by the WHO. This limitation is partially due to the fact that Alere’s marketing strategy does not include China, making it impossible to access their tests. Furthermore, our study focused solely on the HIV-negative population and did not conduct lymphocyte-based stratification in HIV-positive patients. Future studies are warranted to evaluate lymphocyte stratification in HIV-positive populations to ensure broader applicability and validate the potential of this approach across diverse patient groups.

The strengths of our study are as follows: the study was conducted in China which is among the high-burden countries. Therefore, the results of this study are eligible to be referenced in similar settings. Our study studied the impact of lymphocytes on both diagnostic efficiency and cost-effectiveness. High performance and low price might push the change of algorithm of TB diagnosis. Our study validates the efficacy of the new AIMLAM kit for the diagnosis of tuberculosis in an HIV-negative population.

## 5 Conclusion

New chemiluminescence-based LAM tests have the potential to diagnose all people with presumptive TB irrespective of HIV condition. After stratifying patients using lymphocyte cell counting, the sensitivity of AIMLAM significantly increased among patients with low lymphocyte counts, and the cost of detecting individual TB cases decreased significantly. Thanks to the accessibility of CBC and the high sensitivity of LAM testing, this provides a new strategy for the diagnosis of ATB, especially in resource-limited areas.

## Data Availability

The raw data supporting the conclusions of this article will be made available by the authors, without undue reservation.
