# Supplementary material for: A new strategy improving TB diagnosis: stratified urine LAM test based on lymphocyte counts
Source: Front Cell Infect Microbiol. 2025 Feb 20;15:1498651. doi: 10.3389/fcimb.2025.1498651 (PMC11882560; doi:10.3389/fcimb.2025.1498651)
Supplement: Supplementary file 1 [file DataSheet1.docx]

**Supplementary Appendix for:**

**A New Strategy Improving TB Diagnosis: Stratified Urine LAM Test Based on Lymphocyte Counts**

**Table of Contents**

[Supplemental Table 1. Demographics of participants. 2](#_Toc166509893)

[Supplemental Table 2 Clinical characters of LAM+ and LAM- 4](#_Toc166509894)

[Supplemental Figure 1 Diagnostic performance compared to CRS 7](#_Toc166509895)

[Supplemental Figure 2. Fagan nomogram 8](#_Toc166509896)

# Supplemental Table 1. Demographics of participants.

| Characteristic | Patients, No. (%) | | | | *P* value |  |
| --- | --- | --- | --- | --- | --- | --- |
|  | Total(N=248) | Definite TB (N=113) | Possible TB (N=53) | Not TB(N=82) |  |  |
| Sex |  |  |  |  | 0.017 |  |
| male | 131(52.82%) | 67(59.29%) | 19(35.85%) | 45(54.88%) |  |  |
| female | 117(47.18%) | 46(40.71%) | 34(64.15%) | 37(45.12%) |  |  |
| Age group, y | |  |  |  | 0.365 |  |
| 30 | 53(21.37%) | 27(23.89%) | 10(18.87%) | 16(19.51%) |  |  |
| 30-60 | 77(31.05%) | 35(30.97%) | 12(22.64%) | 30(36.59%) |  |  |
| 60 | 118(47.58%) | 51(45.13%) | 31(58.49%) | 36(43.90%) |  |  |
| DM | 53(21.37%) | 38(33.63%) | 4(7.55%) | 11(13.41%) | <0.001 |  |
| Hypertension | 39(15.73%) | 17(6.85%) | 7(2.82%) | 15(6.05%) | 0.704 |  |
| CHD | 31(12.50%) | 11(4.44%) | 6(2.42%) | 14(5.65%) | 0.297 |  |
| Tumor | 2(0.81%) | 1(0.40%) | 0(0.00%) | 1(0.40%) | 0.735 |  |
| Auto immunity | 7(2.82%) | 4(3.54%) | 1(1.89%) | 2(2.44%) | 0.809 |  |
| History of TB | 24(9.68%) | 12(10.62%) | 3(5.66%) | 3(3.66%) | 0.159 |  |
| COPD | 4(1.61%) | 1(0.88%) | 1(1.89%) | 2(2.44%) | 0.686 |  |
| WBC, 10^9^/L |  |  |  |  | 0.651 |  |
| <4 | 48(19.35%) | 19(16.81%) | 14(26.42%) | 15(18.29%) |  |  |
| 4-10 | 186(75.00%) | 87(76.99%) | 37(69.81%) | 62(75.61%) |  |  |
| >10 | 14(5.65%) | 7(6.19%) | 2(3.77%) | 5(6.10%) |  |  |
| Neutrophils, 10^9^/L |  |  |  |  | 0.476 |  |
| <1.8 | 49(19.76%) | 23(20.35%) | 13(24.53%) | 13(15.85%) |  |  |
| 1.8-6.3 | 198(79.84%) | 90(79.65%) | 40(75.47%) | 68(82.93%) |  |  |
| >6.3 | 1(0.40%) | 0(0.00%) | 0(0.00%) | 1(1.22%) |  |  |
| Monocytes, 10^9^/L |  |  |  |  | 0.002 |  |
| <0.1 | 1(0.40%) | 0(0.00%) | 0(0.00%) | 1(1.22%) |  |  |
| 0.1-0.6 | 202(81.45%) | 83(73.45%) | 52(98.11%) | 67(81.71%) |  |  |
| >0.6 | 45(18.15%) | 30(26.55%) | 1(1.89%) | 14(17.07%) |  |  |
| Lymphocytes, 10^9^/L |  |  |  |  | <0.001 |  |
| low | 61(24.60%) | 43(38.05%) | 6(11.32%) | 12(14.63%) |  |  |
| normal | 184(74.19%) | 69(61.06%) | 47(88.68%) | 68(82.93%) |  |  |
| high | 3(1.21%) | 1(0.88%) | 0(0.00%) | 2(2.44%) |  |  |
| ESR |  |  |  |  | <0.001 |  |
| normal | 167(67.34%) | 59(52.21%) | 41(77.36%) | 67(81.71%) |  |  |
| high | 81(32.66%) | 54(47.79%) | 12(22.64%) | 15(18.29%) |  |  |
| PCT |  |  |  |  | <0.001 |  |
| normal | 192(77.42%) | 75(66.37%) | 48(90.57%) | 69(84.15%) |  |  |
| high | 56(22.58%) | 38(33.63%) | 5(9.43%) | 13(15.85%) |  |  |
| CRP |  |  |  |  | <0.001 |  |
| normal | 163(65.73%) | 57(50.44%) | 44(83.02%) | 62(75.61%) |  |  |
| high | 85(34.27%) | 56(49.56%) | 9(16.98%) | 20(24.39%) |  |  |

DM: Diabetes mellitus, CHD: chronic heart disease, TB: tuberculosis, COPD: chronic obstructive pulmonary disease, WBC: white blood cell, ESR: erythrocyte sedimentation rate, PCT: procalcitonin; CRP: C-reactive protein.

# Supplemental Table 2 Clinical characters of LAM+ and LAM-

| Characteristic | MRS+, No. (%) | |  | MRS-, No. (%) | |  |
| --- | --- | --- | --- | --- | --- | --- |
|  | LAM - | LAM + | *P* value | LAM - | LAM + | *P* value |
| n | 32 | 81 |  | 129 | 6 |  |
| Lymphocytes |  |  | <0.001 |  |  | 0.929 |
| low | 3(9.38%) | 40(49.38%) |  | 17(13.18%) | 1(16.67%) |  |
| normal | 29(90.63%) | 41(50.62%) |  | 110(85.27%) | 5(83.33%) |  |
| high |  |  |  | 2(1.55%) | 0(0.00%) |  |
| Sex |  |  | 0.663 |  |  | 0.583 |
| female | 12(37.50%) | 34(41.98%) |  | 69(53.49%) | 2(33.33%) |  |
| male | 20(62.50%) | 47(58.02%) |  | 60(46.51%) | 4(66.67%) |  |
| Age group, y |  |  | 0.195 |  |  | 0.381 |
| 30 | 11(34.38%) | 16(19.75%) |  | 26(20.16%) | 0(0.00%) |  |
| 30-60 | 7(21.88%) | 28(34.57%) |  | 64(49.61%) | 3(50.00%) |  |
| 60 | 14(43.75%) | 37(45.68%) |  | 39(30.23%) | 3(50.00%) |  |
| DM | 6(18.75%) | 17(20.99%) | 0.790 | 15(11.63%) | 0(0.00%) | 1 |
| Hypertension | 4(12.50%) | 13(16.05%) | 0.854 | 21(16.28%) | 1(16.67%) | 1 |
| CHD | 2(6.25%) | 9(11.11%) | 0.665 | (0.00%) | (0.00%) | 0.588 |
| COPD | 0(0.00%) | 2(2.47%) | 1 | (0.00%) | (0.00%) | 1 |
| Tumor | 0(0.00%) | 1(1.23%) | 1 | 1(0.78%) | 0(0.00%) | 1 |
| Autoimmunity | 0(0.00%) | 2(2.47%) | 1 | 0(0.00%) | 0(0.00%) |  |
| History of TB | 5(15.63%) | 7(8.64%) |  | 6(4.65%) | 0(0.00%) | 1 |
| WBC, 10^9^/L |  |  | 0.226 |  |  | 0.679 |
| <4 | 6(18.75%) | 13(16.05%) |  | 27(20.93%) | 2(33.33%) |  |
| 4-10 | 26(81.25%) | 61(75.31%) |  | 95(73.64%) | 4(66.67%) |  |
| >10 | 0(0.00%) | 7(8.64%) |  | 7(5.43%) | 0(0.00%) |  |
| Neutrophils, 10^9^/L |  |  | 0.019 |  |  | 0.456 |
| <1.8 | 2(6.25%) | 21(25.93%) |  | 26(20.16%) | 0(0.00%) |  |
| 1.8-6.3 | 30(93.75%) | 69(85.19%) |  | 102(79.07%) | 6(100.00%) |  |
| >6.3 | 0 | 0 |  | 1(0.78%) | 0(0.00%) |  |
| Monocytes, 10^9^/L |  |  | 0.815 |  |  | 0.656 |
| <0.1 | 0 | 0 |  | 1(0.78%) | 0(0.00%) |  |
| 0.1-0.6 | 24(75.00%) | 57(70.37%) |  | 113(87.60%) | 6(100.00%) |  |
| >0.6 | 8(25.00%) | 22(27.16%) |  | 15(11.63%) | 0(0.00%) |  |
| ESR |  |  | 0.009 |  |  | 0.754 |
| normal | 23(71.88%) | 36(44.44%) |  | 25(19.38%) | 2(33.33%) |  |
| high | 9(28.13%) | 45(55.56%) |  | 104(80.62%) | 4(66.67%) |  |
| PCT |  |  | 0.011 |  |  | 1 |
| normal | 27(84.38%) | 48(59.26%) |  | 18(13.95%) | 0(0.00%) |  |
| high | 5(15.63%) | 33(40.74%) |  | 111(86.05%) | 6(100.00%) |  |
| CRP |  |  | 0.004 |  |  | 0.83 |
| normal | 23(71.88%) | 34(41.98%) |  | 27(20.93%) | 2(33.33%) |  |
| high | 9(28.13%) | 47(58.02%) |  | 102(79.07%) | 4(66.67%) |  |

MRS: micrologically reference standard, DM: Diabetes mellitus, CHD: chronic heart disease, ; TB,: tuberculosis,; COPD:, chronic obstructive pulmonary disease, WBC: white blood cell, ESR: . erythrocyte sedimentation rate, PCT: procalcitonin; CRP: C-reactive protein.


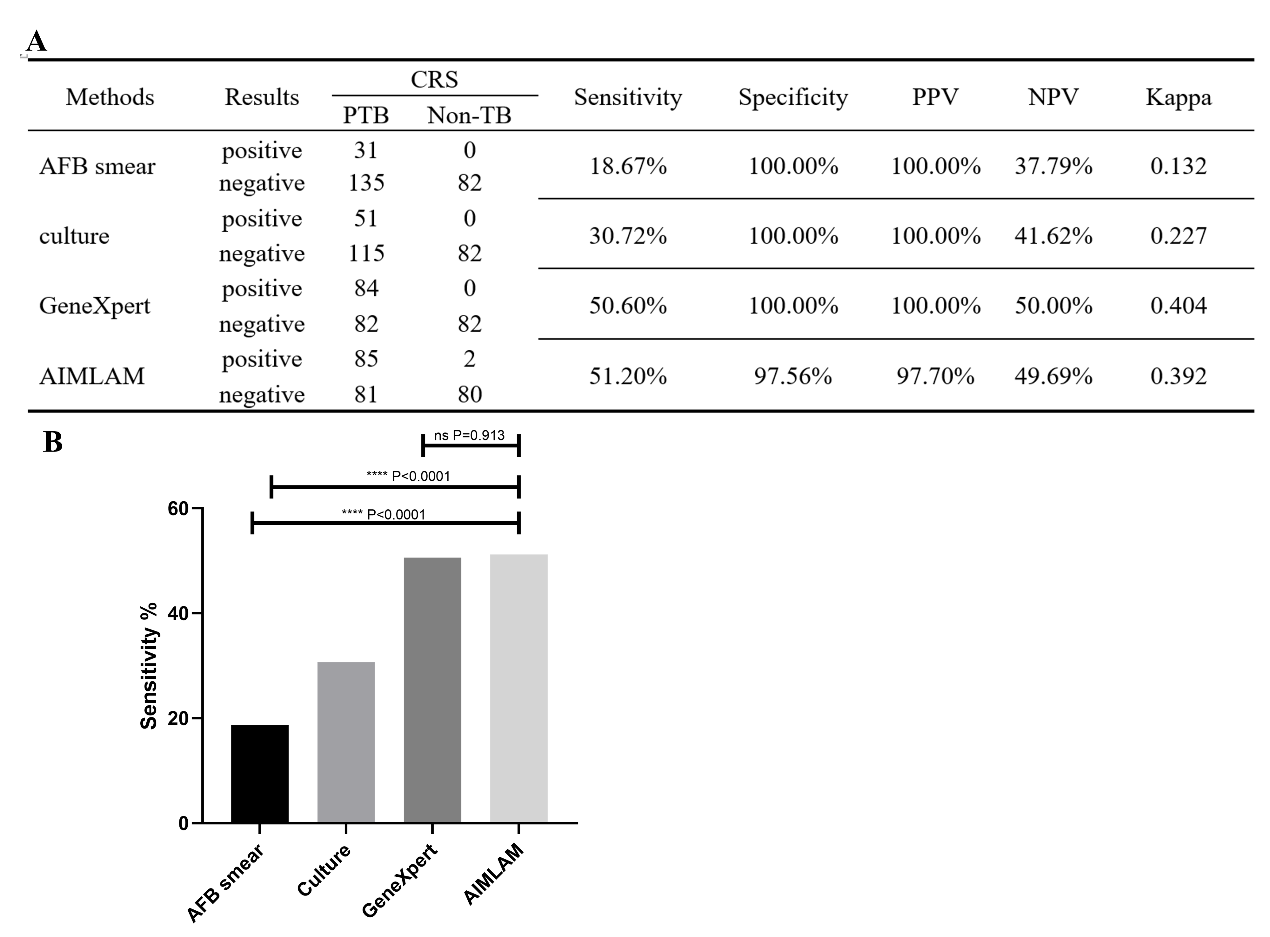


Supplemental Figure 1 Diagnostic performance compared to CRS; (A) Diagnostic performance of four methods compared to CRS; (B) Sensitivity difference analysis of four methods based on CRS. AFB: Acid-Fast Bacillus LAM: lipoarabinomannan, CRS: composite reference standard, PPV: positive predictive value, NPV: negative predictive value.

Sensitivity = True Positive Cases / (True Positive Cases + False Negative Cases) × 100%;

Specificity = True Negative Cases / (True Negative Cases +False Positive Cases) × 100%;

PPV = True Positive Cases / (True Positive Cases + False Positive Cases) × 100%;

NPV = True Negative Cases / (True Negative Cases + False Negative Cases) × 100%;


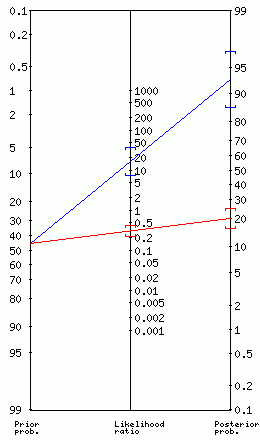


# Supplemental Figure 1. Fagan nomogram

POSITIVE TEST (blue line):

Positive Likelihood ratio: 16%

95% confidence interval: [7.32%,36%]

Posterior probability (odds): 93% (13.4)

95% confidence interval: [86%,97%]

NEGATIVE TEST (red line):

Negative Likelihood ratio: 0.30%

95% confidence interval: [0.22%,0.40%]

Posterior probability (odds): 20% (0.3)

95% confidence interval: [16%,25%]
